# Transcriptional regulation of the *Pseudomonas aeruginosa* iron-sulfur cluster assembly pathway by binding of IscR to multiple sites

**DOI:** 10.1371/journal.pone.0218385

**Published:** 2019-06-28

**Authors:** Kritsakorn Saninjuk, Adisak Romsang, Jintana Duang-nkern, Paiboon Vattanaviboon, Skorn Mongkolsuk

**Affiliations:** 1 Department of Biotechnology, Faculty of Science, Mahidol University, Bangkok, Thailand; 2 Center for Emerging Bacterial Infections, Faculty of Science, Mahidol University, Bangkok, Thailand; 3 Laboratory of Biotechnology, Chulabhorn Research Institute, Bangkok, Thailand; 4 Program in Applied Biological Sciences: Environmental Health, Chulabhorn Graduate Institute, Chulabhorn Royal Academy, Bangkok, Thailand; 5 Center of Excellence on Environmental Health and Toxicology, EHT, Ministry of Education, Bangkok, Thailand; East Carolina University Brody School of Medicine, UNITED STATES

## Abstract

Iron-sulfur ([Fe-S]) cluster proteins have essential functions in many biological processes. [Fe-S] homeostasis is crucial for bacterial survival under a wide range of environmental conditions. IscR is a global transcriptional regulator in *Pseudomonas aeruginosa*; it has been shown to regulate genes involved in [Fe-S] cluster biosynthesis, iron homeostasis, resistance to oxidants, and pathogenicity. Many aspects of the IscR transcriptional regulatory mechanism differ from those of other well-studied systems. This study demonstrates the mechanisms of IscR Type-1 binding to its target sites that mediate the repression of gene expression at the *isc* operon, *nfuA*, and *tpx*. The analysis of IscR binding to multiple binding sites in the promoter region of the *isc* operon reveals that IscR first binds to the high-affinity site B followed by binding to the low-affinity site A. The results of *in vitro* IscR binding assays and *in vivo* analysis of IscR-mediated repression of gene expression support the role of site B as the primary site, while site A has only a minor role in the efficiency of IscR repression of gene expression. Ligation of an [Fe-S] cluster to IscR is required for the binding of IscR to target sites and *in vivo* repression and stress-induced gene expression. Analysis of Type-1 sites in many bacteria, including *P*. *aeruginosa*, indicates that the first and the last three AT-rich bases were among the most highly conserved bases within all analyzed Type-1 sites. Herein, we first propose the putative sequence of *P*. *aeruginosa* IscR Type-1 binding motif as 5’AWWSSYRMNNWWWTNNNWSGGNYWW3’. This can benefit further studies in the identification of novel genes under the IscR regulon and the regulatory mechanism model of *P*. *aeruginosa* IscR as it contributes to the roles of an [Fe-S] cluster in several biologically important cellular activities.

## Introduction

*Pseudomonas aeruginosa* is an important environmental and human pathogenic Gram-negative bacterium. During the infection process, bacteria confront oxidative stress generated by host defense mechanisms. *P*. *aeruginosa* contains a plethora of antioxidant enzymes/proteins and reactive oxygen species (ROS)-sequestering compounds that contribute to the protection against oxidative stress and are important for bacterial proliferation and successful infections [1–6].

An iron-sulfur ([Fe-S]) cluster is believed to be an ancient type of protein cofactor, and its biosynthesis process appears to be remarkably conserved in most organisms [7, 8]. [Fe-S] cluster-containing proteins are required for many biological processes, including biosynthesis pathway activities, respiration, central metabolism, photosynthesis, nitrogen fixation, DNA repair, RNA modification, and gene regulation, across all domains of life [9, 10]. However, active [Fe-S] clusters are rapidly damaged by univalent oxidants, and the loss of an electron destabilizes the cluster, causing it to release its catalytic iron atom and initially converting the cluster to an inactive [3Fe-4S]^1+^ form, often leading to defective proteins [11, 12]. Continued exposure to oxidants leads to further oxidation and iron release.

Three independent [Fe-S] cluster biogenetic systems, named ISC (iron-sulfur cluster), SUF (mobilization of sulfur), and NIF (nitrogen fixation), have been found in prokaryotes. The ISC and SUF are general [Fe-S] cluster biogenesis systems, whereas the NIF system is specific for the assembly and insertion of [Fe-S] clusters in nitrogenase, the enzyme involving in nitrogen fixation [7]. Bacterial genome analyses reveal variations in the number and type of these systems. *Azotobacter vinelandii* has both *nif* and *isc* operons whereas *Escherichia coli* and *Therminola potens*, have both the ISC and SUF systems. Some bacteria contain only one system, such as the ISC system in the human pathogen *P*. *aeruginosa* [13], and the SUF system in the Gram-negative plant pathogen *Xanthomonas campestris* pv. campestris [14]. All three [Fe-S] biosynthetic systems require a cysteine desulfurase (NifS, IscS, and SufS), which allows the utilization of L-cysteine as a source of sulfur atoms. The scaffold proteins (IscU, IscA, NifU, SufU, and SufA) provide an intermediate assembly site for [Fe-S] cluster precursors, from which these clusters can be donated to apo-proteins [10, 15, 16]. In *Escherichia coli*, the transcriptional regulator IscR is essential for [Fe-S] cluster homeostasis regulation, but additional regulators such as Fur, OxyR, and possibly NsrR are also involved [10, 17]. IscR is a [2Fe-2S] transcriptional regulator encoded by the first gene of the *iscRSUA-hscBA-fdx* operon. Under normal condition, the ISC system is the major cluster biogenesis, which is considered as a housekeeping [Fe-S] cluster biogenesis pathway [18]. During stress conditions, the active clusters are damaged, leading to depression of ISC and activated SUF systems via apo-IscR. Thus, the ISC and SUF systems display some functional redundancy in *E*. *coli* [19]. Mutagenesis studies have shown that IscR contains three cysteine residues (C92, C98, and C104) and a histidine residue (H107) that are essential for [2Fe-2S] ligation [20, 21]. Previous studies have identified two types of IscR binding sites, Type-1 (5’ATASYYGACTRWWWYAGTCRRSTAT3’) and Type-2 (5’AWARCCCYTSnGTTTGMnGKKKTKWA3’) [22]. It was demonstrated in *E*. *coli* that holo-IscR, containing [2Fe-2S], binds the Type-1 site with a higher affinity than apo-IscR, whereas both the holo- and apo-form bind similarly to the Type-2 site [22, 23].

In the *P*. *aeruginosa* genome, the complete set of genes encoding the SUF machinery was not identified, and only the ISC system was presented [18]. The *iscRSUA-hscBA-fdx2-iscX* operon in *P*. *aeruginosa* was upregulated under oxidative stress, and IscR acts as a sensor of cellular [Fe-S] levels and a global transcription regulator for [Fe-S] biogenesis under both physiological and stressful conditions [13]. Moreover, IscR senses [Fe-S] and directly represses or activates the transcription of genes affecting many physiological pathways, including iron metabolism, type-3 secretion, and bacterial virulence [14, 24–27]. *P*. *aeruginosa* IscR was described, through an *in vitro* binding assay and gene expression analysis, as a repressor of *tpx*, a gene encoding thiol peroxidase, which plays a role in hydrogen peroxide resistance [5] and of *nfuA*, in which its gene product functions in [Fe-S] cluster delivery and is required for sustaining growth under stress and anaerobic conditions and maintaining bacterial virulence [25]. The C92, C98, C104, and H107 residues in *P*. *aeruginosa* IscR were shown to be involved in [Fe-S] cluster ligation; however, their roles in the transcriptional regulatory mechanism remain unknown. In this study, two Type-1 binding sites of IscR were found on the promoter of the *isc* operon in *P*. *aeruginosa*, and the stepwise modulations of the IscR on the regulatory mechanisms were investigated by both *in vitro* and *in vivo* experiments. In addition, the single Type-1 IscR binding motif and the regulatory mechanism of IscR regulated target genes, *tpx* and *nfuA*, were evidently elucidated. Ultimately, the *P*. *aeruginosa* IscR Type-1 sequence logo was refined and could be useful for the identification of novel IscR Type-1 regulated genes.

## Materials and methods

### Bacterial strains and growth conditions

All bacterial strains and plasmids used in this study are listed in Table 1. Both *E*. *coli* and *P*. *aeruginosa* (PAO1) strains were aerobically cultivated in Luria-Bertani broth (LB) at 37°C. The media for *E*. *coli* and PAO1 growth were supplemented with 100 μg ml^-1^ ampicillin (Ap) or 15 μg ml^-1^ gentamicin (Gm) and 200 μg ml^-1^ carbenicillin (Cb) or 30 μg ml^-1^ Gm, respectively. Exponential phase cells (OD_600nm_ about 0.6, after 3 h of growth) were used in all experiments. All *P*. *aeruginosa* were raised, maintained, and all experiments were conducted following procedure MUSC2018-015 approved by the Committee of Biosafety, Faculty of Science, Mahidol University.

**Table 1:** Strains and plasmids used in this study.

Strain or Plasmid	Relevant characteristic(s)	Source
**Strains**		
*P*. *aeruginosa* PAO1	Wild-type strain	ATCC15692
PAO1/pBBR	PAO1 harboring pBBR1MCS-4	This study
PAO1::Tn7T	PAO1 transposed with pUC18-mini-Tn7T-Gm-LAC	This study
Δ*iscR*	PAO1 Δ*iscR* mutant	[13]
Δ*iscR*::Tn7T	Δ*iscR* mutant transposed with pUC18-mini-Tn7T-Gm-LAC	[13]
Δ*iscR*/pBBR	Δ*iscR* mutant harboring pBBR1MSC-4	[13]
Δ*iscR*/pBBR-*iscR*	Δ*iscR* mutant harboring pBBR-*iscR*	[13]
Δ*iscR*/pBBR-*iscR-*3CA	Δ*iscR* mutant harboring pBBR-*iscR-*C92, 98, 104A	This study
**Plasmids**		
pUC18-mini-Tn7T-Gm-LAC	pUC18 containing mini-Tn7T::P_*lac*_ site, Ap^r^, Gm^r^	[28]
pUC18-mini-Tn7T-Gm-P_*iscR*_-NP	pUC18 containing native *iscR* promoter	This study
pUC18-mini-Tn7T-Gm-P_*iscR*_-MU1	pUC18 containing MU1 mutagenized *iscR* promoter	This study
pUC18-mini-Tn7T-Gm-P_*iscR*_-MU2	pUC18 containing MU2 mutagenized *iscR* promoter	This study
pUC18-mini-Tn7T-Gm-P_*iscR*_-MU3	pUC18 containing MU3 mutagenized *iscR* promoter	This study
pBBR1MCS-4	Medium-copy-number expression vector, Ap^r^	[29]
pBBR-*iscR*	pBBR1MCS-4 containing *iscR*	[13]
pBBR-*iscR-*3CA	pBBR1MCS-4 containing *iscR-*C92, 98, 104A	This study
pET-*iscR*	pETBlue-2 containing full-length *iscR* expression, Ap^r^	[13]
pET-*iscR-*C92A	pET-*iscR* with C92A mutagenesis	This study
pET-*iscR-*C98A	pET-*iscR* with C98A mutagenesis	This study
pET-*iscR-*C104A	pET-*iscR* with C104A mutagenesis	This study
pET-*iscR-*H107A	pET-*iscR* with H107A mutagenesis	This study
pET-*iscR-*C111A	pET-*iscR* with C111A mutagenesis	This study
pET-*iscR-*3CA	pET-*iscR* with C92, 98, 104A mutagenesis	This study
pCM351	Vector containing the *loxP*-Gm^r^-*loxP* region, Gm^r^	[30]

### Molecular techniques

General molecular techniques including DNA and RNA preparation, DNA cloning, PCR amplification, Southern and Northern analyzes, and *E*. *coli* transformation were performed using standard protocols [31]. Transformation of plasmids into *P*. *aeruginosa* strains was carried out using electroporation as previously described [32]. The oligonucleotide primers used are listed in Table 2.

**Table 2:** List of primers used in this study.

Primer	Sequence (5’→3’)	Purpose
M13F	GTAAAAGGACGGCCAGT	Universal upstream primer
M13R	GAAACAGCTATGACCATG	Universal downstream primer
pETBlueUP	TCACGACGTTGTAAAACGA	pETBlue-2 vector system upstream primer
BT3612	GAAGATTTCGCCGGAGTCAA	Forward primer for *iscR* promoter analysis
BT3613	GCGTTCGGAGATATCCGGCCAG	Reverse primer for *iscR* promoter analysis
BT4303	CTCCCCCTGAGTAATTTGATC	Site-directed mutagenesis of *iscR* promoter (MU1)
BT4304	CTCAGGGGGAGGCAAATTCAG	Site-directed mutagenesis of *iscR* promoter (MU1)
BT4305	TTCCCGTTGACCTAATTACTC	Site-directed mutagenesis of *iscR* promoter (MU2)
BT4306	AGGTCAACGGGAAGACCGATC	Site-directed mutagenesis of *iscR* promoter (MU2)
EBI369	AATTACTCGCCCCCGAGCATAAT	Site-directed mutagenesis of *iscR* promoter (MU3)
EBI370	ATTATGCTCGGGGGCGAGTAATT	Site-directed mutagenesis of *iscR* promoter (MU3)
EBI6	CGTCTGACCACCAAAGGCCGCTACGC	Forward primer for *iscR* protein expression
EBI7	CCGCTCGAGGTCGATGGCGGACGCTTCAATC	Reverse primer for *iscR* protein expression
BT2879	AACCGCTACGAGAACCTC	Forward primer for *nfuA* promoter analysis
BT3291	CGAGACGAAGAGGTCGTT	Reverse primer for *nfuA* promoter analysis
BT7215	GACCCG GGAGTATTTCGCCA	Forward primer for *tpx* promoter analysis
BT3590	GCCTTTCTGCGGGAGCT	Reverse primer for *tpx* promoter analysis
BT2841	ACCATCCCGCAGCCCTG	Forward primer for *nfuA* expression
BT2860	ACCGCCATCGCCCTGAAG	Reverse primer for *nfuA* expression
BT2647	GAAGGATCAACGCAATGG	Forward primer for *tpx* expression
BT2649	ACCACGGTGTTGGCCAGC	Reverse primer for *tpx* expression

### Site-directed mutagenesis constructions

PCR-based site-directed mutagenesis was performed to convert three cysteine residues (C92, C98, and C104) of the IscR to alanine (A) residues using pBBR-*iscR* expression plasmid [13] as DNA template, generating a IscR-3CA mutation as previously described [33] and using a primer pair as shown in Table 2. The PCR products containing mutated sequences were cloned into an expression vector, pBBR1MCS-4 [29]. The mutations were verified by DNA sequencing.

### Purification of IscR wild-type and IscR variants

6x His-tagged IscR from *P*. *aeruginosa* was purified using the pETBlue-2 expression system as previously described [13]. IscR variant proteins, IscR-C92A, IscR-C98A, IscR-C104A, IscR-H107A, IscR-C111A, and IscR-3CA were purified as previously described [5, 13], except that the primers and the PCR templates were used in the PCR-based site-directed mutagenesis as indicated in Table 2. The purity of IscR protein was more than 90%, as determined by a major band corresponding to the 18.7 kDa protein observed in SDS-PAGE. The UV-visible spectroscopy analysis of various mutated IscR variants were carried out using a Shimadzu UV-1800 spectrophotometer to analyze the presence of an [Fe-S] cluster ligated IscR as previously described [3, 13]. The His-tagged IscR-3CA protein was purified, and UV-visible spectroscopy scanning analysis showed an absence of an absorbance peak at 420 nm suggesting a deficiency in the [2Fe-2S] cluster ligation of the IscR mutant protein (S1 File).

### Gel mobility shift assay

Gel mobility shift assays were performed by using a labeled probe containing either native or mutagenic *iscR*-promoters that were amplified using ^32^P-labeled BT3612 and BT3613 primers and native or mutagenized *iscR*-promoters as DNA templates as previously described [4, 25, 34]. Binding reactions were conducted using 3 fmol of labeled probe in 25 μl of reaction buffer containing 20 mM Tris-HCl (pH 8.0), 50 mM KCl, 4 mM MgCl_2_, 0.5 mM EDTA, 0.02 mg ml^-1^ bovine serum albumin (BSA), 5 mM dithiothreitol (DTT), 10% (v/v) glycerol, and 200 ng of poly(dI-dC). Various amounts of purified IscR proteins were added, and the reaction mixture was incubated at 25°C for 20 min. Protein-DNA complexes were separated by electrophoresis on a 7% nondenaturing polyacrylamide gel in 0.25x Tris-borate-EDTA (TBE) buffer at 4°C and visualized by exposure to X-ray film.

### DNaseI footprinting analysis

Experiments were performed as previously described [35]. Essentially, the binding reactions were incubated at room temperature for 30 min before digestion in a total reaction volume of 100 μl with 0.2 units of DNaseI (Promega), 5 mM CaCl_2_, 10 mM MgCl_2_, and 1 μg ml^-1^ salmon sperm at 37°C for 25 s. The DNaseI digestion reaction was stopped by adding 700 μl of stop solution (649 μl of absolute ethanol, 50 μl of 3 M sodium acetate, and 1 μl of 1 mg ml^-1^ yeast tRNA). The digested DNA products were harvested and resuspended in Milli-Q water and the DNA fragments were analyzed on a 6% denaturing polyacrylamide gel in 1x TBE buffer. A sequence ladder was generated by using an *fmol* DNA sequencing system (Promega). The promoter fragments were prepared by PCR amplification using ^32^P end-labeled forward primer and unlabeled reverse primer (BT3612 [5’GAAGATTTCGCCGGAGTCAA3’] and BT3613 [5’GCGTTCGGAGATATCCGGCCAG3’] for P_*iscR*_, BT2879 [5’AACCGCTACGAGAACCTC3’] and BT3291 [5’CGAGACGAAGAGGTCGTT3’] for P_*nfuA*_, and BT7215 [5’GACCCGGGAGTATTTCGCCA3’] and BT3590 [5’GCCTTTCTGCGGGAGCT3’] for P_*tpx*_) and each promoter fragment as DNA template. The sequence ladder was generated using DNA promoter fragment as template.

### *iscR* promoter-*lacZ* fusion

The PAO1 strain carrying *iscR* promoter-*lacZ* fusion was constructed using mini-Tn7 transposon. The 294-bp putative promoter region of *iscR* was PCR amplified from the PAO1 genomic DNA using primers BT3612 and BT3613 (see Table 2). The PCR product was cloned into pUC18 at SmaI site before the promoter fragment was subcloned in both orientations into a *lacZ* transcriptional fusion vector pUC18-mini-Tn7T-Gm-*lacZ* [28] cut with KpnI and PstI to generate pMini-Tn7T-P_*iscR*_ and pMini-Tn7T-P_*iscR*_C (for the promoter fragment in reverse orientation was fused to *lacZ* as a control). The recombinant plasmids and pTNS2 [28], which encodes TnsABCD transposase enzyme that is required for *att*Tn7 site-specific transposition, were introduced into *P*. *aeruginosa* strains and insertion of the mini-Tn7 containing *iscR* promoter-*lacZ* fusion on the bacterial chromosome was confirmed as previously described [28].

### Site-directed mutagenesis of the *iscR* promoter sequence

Site-directed mutagenesis was performed to mutate the putative IscR binding sites on the *iscR* promoter from 5’AATCCTGAGTAATTTGATCGGTCTT3’ to 5’CCCCCTGAGTAATTTGATCGGTCTT3’ for site A mutagenesis (MU1) and from 5’ATAGTTGACCTAATTACTCGGATAA3’ to 5’CCCGTTGACCTAATTACTCGGATAA3’ for 5’-end site B mutagenesis (MU2) and to 5’ATAGTTGACCTAATTACTCGCCCCC3’ for 3’-end site B mutagenesis (MU3) by using PCR-based mutagenesis as previously described [25]. The sequences of mutagenic forward and reverse primers for site-directed mutagenesis at IscR binding site A, BT4303 and BT4304, at the 5’-end of IscR binding site B, BT4305 and BT4306, and at the 3’-end of IscR binding site B, EBI369 and EBI370, are shown in Table 2. PCR amplification was performed using pMini-Tn7T-P_*iscR*_ as template. The PCR products were cloned into pUC18-mini-Tn7T-Gm-*lacZ*, generating pMini-Tn7T-_P*iscR*_-MU1, pMini-Tn7T-P_*iscR*_-MU2, and pMini-Tn7T-P_*iscR*_-MU3, respectively. The mutated promoters were verified by DNA sequencing.

### β-galactosidase activity assay

β-galactosidase activity assay was performed by colorimetric monitored using an o-nitrophenyl-β-D-galactopyranoside (ONPG) [36]. One unit (U) of β-galactosidase is expressed as the amount of enzyme capable of hydrolyzing 1 nmole of ONPG per minute at the assay condition. The unit of β-galactosidase is divided by the total protein obtained using Bradford assay. Data were obtained from three independent experiments. The unit of β-galactosidase is divided by the total protein obtained using Bradford assay. Data were obtained from three independent experiments.

### RNA extraction and qRT-PCR

RNA extraction and qRT-PCR was performed as previously described [2]. In brief, total RNA was extracted from either between uninduced and stress induced cultures in *P*. *aeruginosa* strains by hot acidic phenol method [31]. After DNase I treatment, the RNA was reversed transcribed and 10 ng cDNA was added into a KAPA SYBR FAST qPCR kit containing gene specific primer pairs (BT2841 and BT2860 for *nfuA* [37]; BT3186 and BT3187 for *iscR* [13]; BT2647 and BT2649 for *tpx* [5]; BT2781 and BT2782 for *16S rRNA* as an internal control). The reactions were applied to Applied Biosystems StepOnePlus under the following conditions: denaturation at 95°C for 10 s, annealing at 60°C for 30 s, and extension at 60°C for 30 s, for 40 cycles. Relative expression was calculated using STEPONE software v2.1 and expressed as folds of expression relative to the level of PAO1 wild type grown under uninduced condition. The fold change of gene expressions were calculated in comparison to a reference gene using *16S rRNA*. The data were obtained from at least three biologically independent experiments and were expressed by as the means ± standard deviations (SD).

### Statistical analysis

The significance of differences between strains, cultured conditions, or changes of expression level was statistically determined using one-way ANOVA with Bonferroni correction. The *p* < 0.05 is considered statistically significant difference.

## Results and discussion

**Fig 1:**
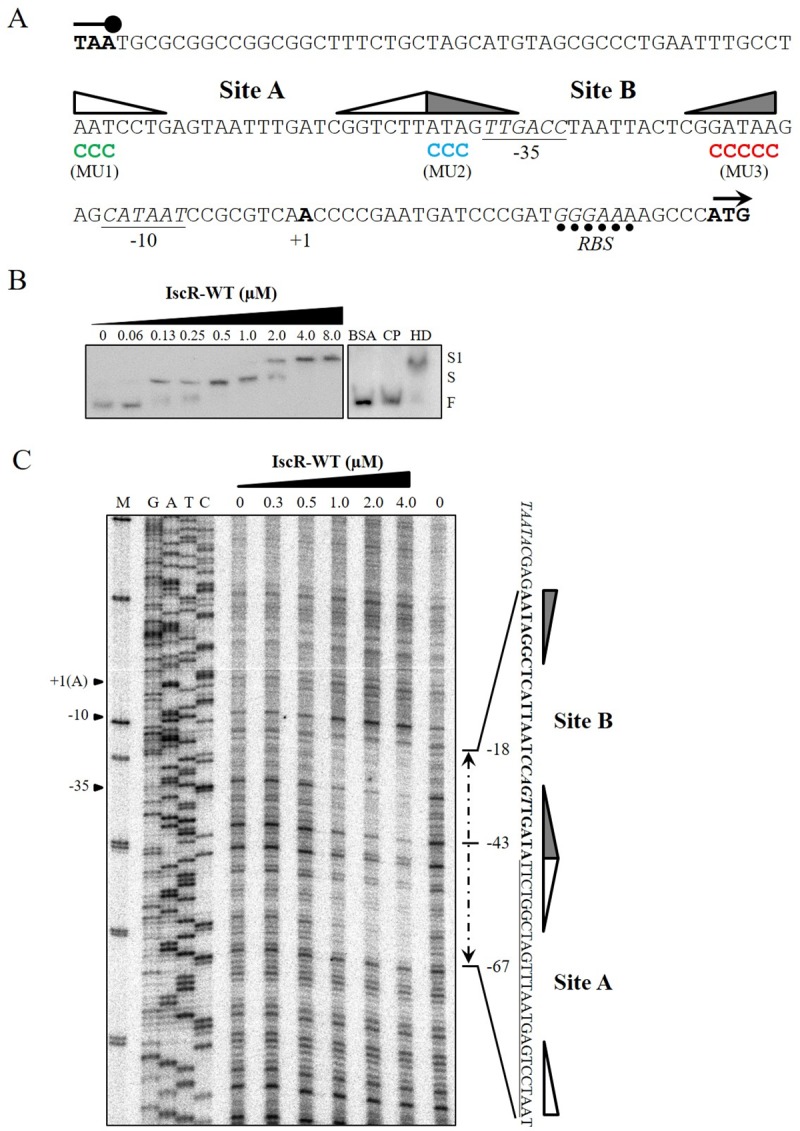
Characterization of the IscR-regulated promoter of *P*. *aeruginosa isc* operon. (A) The promoter architecture of the *isc* operon showing the dual putative IscR binding sites, A and B [13, 18]. The positions of mutagenized IscR binding sites are shown with green, blue, and red fonts for MU1, MU2, and MU3, respectively. Underline and italic font represent the -35 and -10 promoter motifs and ribosome binding site (RBS), respectively. (B) EMSA was performed using the *iscR* promoter fragment and increasing concentrations of purified IscR-WT protein. Two levels of the shifted bands of the protein-DNA complexes were detected. S and S1 indicate shifted and supershifted bands, respectively. F represents the free probe. BSA indicates unrelated protein. CP and HD represent the addition of unlabeled *iscR* promoter and heterologous DNA (pUC18 plasmid), respectively, to the binding reaction mixtures containing 4 μM IscR. (C) The DNaseI footprinting assay was conducted using the *iscR* promoter fragment and purified IscR-WT protein. Two tandem IscR binding sites (site A, underlined sequence and site B, bold sequence) covering the RNA polymerase binding motifs (-35 and -10 regions) are shown. G, A, T, and C represent DNA sequence ladders, and M is the DNA marker.

In our previous gene expression study, we determined that IscR functions as a transcriptional repressor on its own promoter, which supports the regulation of the *iscR* and the *isc* operon [38]. Here, the mechanism of IscR as a repressor of the *isc* operon was investigated. Based on computational analysis of an upstream sequence of the PAO1 *iscR* transcriptional start site (+1), we have proposed two putative IscR binding motifs denoted as site A (5’AATCCTGAGTAATTTGATCGGTCTT3’) and site B (5’ATAGTTGACCTAATTACTCGGATAA3’) located at positions -43 to -67 and -18 to -42, respectively ([13] and Fig 1A). Site A and site B binding motifs had 68% and 76% identity with the consensus sequence for the *E*. *coli* IscR Type-1 binding motif (5’ATASYYGACTRWWWYAGTCRRSTAT3’), respectively [19, 22]. The 5’ AT sequences of each site are among the most highly conserved bases within the Type-1 site [21, 22]. The IscR Type-1 binding site B is located between the -35 and -10 regions of the *iscR* promoter motifs, while site A is located upstream of the -35 regions of the promoter motif (Fig 1A). The binding of IscR to site A and/or site B would impede the binding of RNA polymerase to the promoter, leading to transcriptional repression of the *isc* operon. The role of these putative IscR Type-1 binding sites within the *iscR* promoter region and the requirement of the [Fe-S] cluster ligated IscR were further investigated in this study.

### Multiple Type-1 sites on an *isc* promoter for [Fe-S]-IscR binding

To characterize the multiple IscR binding sites on the *iscR* promoter region, a gel electrophoresis mobility shift assay (EMSA) was performed using purified IscR and the promoter fragments as previously described [4, 25, 34]. The results in Fig 1B showed that purified IscR was able to bind to the *iscR* promoter fragment. At low concentrations (0.13 μM to 1 μM), purified IscR bound to the promoter fragment, giving a single shifted band of the protein-DNA complex; however, at 2 μM IscR, a second (supershifted) binding product could be detected (Fig 1B). When high concentrations (4 μM to 8 μM) of IscR were used in the binding reactions, only the supershifted band was present (Fig 1B). The presence of a supershifted binding fragment at high concentrations of IscR represented either binding of IscR to multiple binding motifs on the *iscR* promoter region or the formation of an oligomeric IscR protein complex on the DNA probe at high concentrations of IscR. The binding specificity of IscR was demonstrated by the ability of the unlabeled *iscR* promoter fragment to compete with the binding of the probe, whereas excess heterologous DNA (pUC18 plasmid) did not interfere with the binding of the probe. Additionally, an excess amount of unrelated protein (2.5 μg ml^-1^ BSA) did not interfere with the binding of IscR to the labeled probe (Fig 1B). Thus, IscR directly bound to the *iscR* promoter.

To clarify the role of the two putative IscR binding motifs in the *iscR* promoter region, DNaseI footprinting analysis was performed using purified IscR and the promoter fragments. The results are shown in Fig 1C. The DNaseI protected regions could be detected at an IscR concentration of 1 μM (Fig 1C). The binding of IscR to the promoter fragment produced an atypically long protected region covering the positions from -18 to -67 on the *iscR* promoter, suggesting the possibility of multiple connected IscR binding motifs in this region (Fig 1C). The previously characterized IscR binding sites are usually 20–30 nucleotides long [19, 20, 22]. The DNaseI footprinting results supported the computational analysis of putative IscR binding sites indicating that the *iscR* promoter region had two putative IscR binding motifs, site A (5’AATCCTGAGTAATTTGATCGGTCTT3’) and site B (5’ATAGTTGACCTAATTACTCGGATAA3’), located between positions -43 to -67 and -18 to -42, respectively (Fig 1A). No other IscR protected region on the promoter fragment could be detected. The results indicated the absence of a putative “site C” in the *P*. *aeruginosa isc* operon promoter region. The minor, nonconserved site C of the IscR binding site has been observed in the *E*. *coli iscR* regulatory regions [19]. Dual IscR Type-1 binding sites could offer a more refined regulation of gene expression that enables rapid and accurate response to changes in the [Fe-S] cluster level.

### Analysis of the mode of IscR binding to multiple binding sites

**Fig 2:**
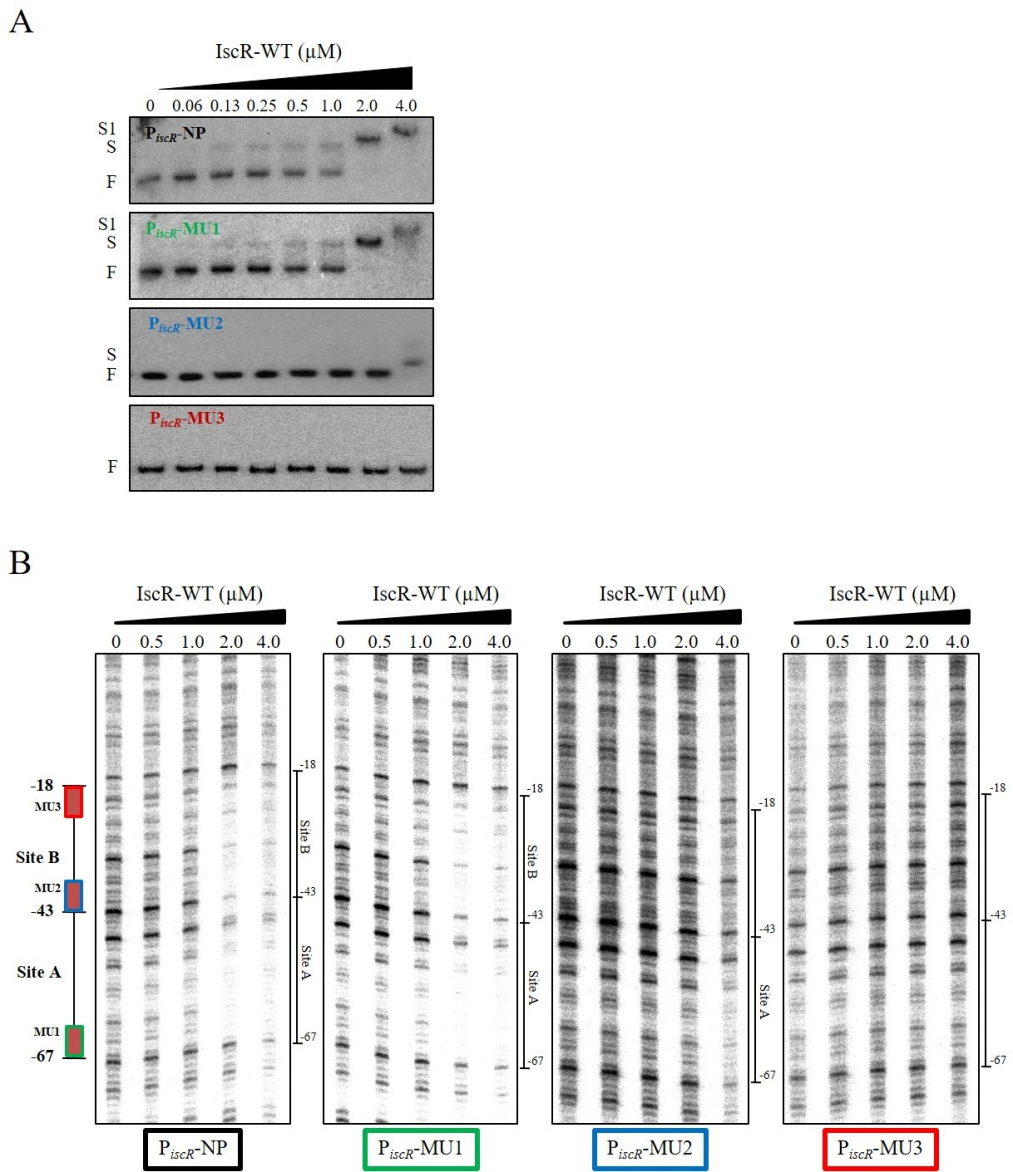
*In vitro* characterization of the mutated *isc* promoter. Site-directed mutagenesis of the putative IscR binding Type-1 sites on the promoter region of the *isc* operon (MU1, MU2, and MU3) was achieved. (A) EMSA and (B) DNaseI footprinting was performed using purified IscR-WT and the mutated *isc* promoter fragments (MU1, MU2, or MU3). F, S, and S1 indicate free probe, shifted, and supershifted bands, respectively.

The above results clearly indicated that there are at least two IscR binding sites on the *iscR* promoter. To clarify the mode and the sequential binding of IscR to these sites, site-directed mutagenesis of the two putative IscR binding motifs on the *iscR* promoter region was performed, and their effects on IscR binding were examined. Both putative binding sites, site A and site B, were highly similar to the *E*. *coli* Type-1 binding motif that contains the AT-conserved nucleotides at both the 5’ and 3’ ends of the motifs. The site-directed mutagenesis of these motifs within the *iscR* promoter fragments created the mutations AAT to CCC at the 5’ end of site A (MU1), ATA to CCC at the 5’ end of site B (MU2) and GATAA to CCCCC at the 3’ end of site B (MU3) that were constructed and used in the binding experiments. These mutations would eliminate IscR binding to these sites based on previously identified *E*. *coli* IscR binding site consensus sequences. An EMSA was performed using purified IscR and mutated MU1, MU2, and MU3 promoter fragments. The EMSA results showed that IscR binding to the native and MU1 promoter fragments had similar binding patterns, showing shifted bands at IscR concentrations ranging from 0.13 μM to 1 μM and the supershifted band at higher concentrations (2 to 4 μM) of IscR (Fig 2A). The mutation of the 5’ end of site A did not alter the IscR binding affinity compared to the native promoter. In contrast, no binding of IscR at concentrations ranging from 0.125 μM to 2 μM to the MU2 and MU3 promoter fragments could be detected (Fig 2A). At 4 μM, IscR bound to MU2 promoter fragments, and no supershifted band was observed (Fig 2A). This feature is different from the binding of IscR to the native promoter fragment, as only a supershifted band could be detected at the IscR concentration of 2 μM (Fig 2A). The mutations at nucleotides either at the 5’ or 3’ end of site B eliminated IscR binding to the promoter fragments, supporting the primary role of site B in the binding of IscR to its promoter fragments. Moreover, the binding of IscR to the mutated 5’ end of site B (MU2) could be observed at a high concentration of IscR (4 μM).

Next, the binding characteristics of IscR to the mutagenized promoter fragments were investigated using DNaseI footprinting. The affinity of IscR binding and the DNaseI-protected regions on the *isc* promoter of MU1 and the native promoter fragment were similar (Fig 2B). The binding of IscR produced to a protected region from -18 to -67, encompassing both site A and site B for both native and mutated MU1 promoter fragments (Fig 2B). In contrast, site B mutations MU2 and MU3 of *isc* promoter fragments practically eliminated IscR binding to the site B region. At IscR concentrations ranging from 0.25 μM to 2 μM, no protected regions encompassing sites A and B were observed for both MU2 and MU3 promoter fragments (Fig 2B). Nonetheless, at 4 μM IscR, the protected region of the MU2 fragment was detected from -43 to -67, which encompassed only the site A region (Fig 2B). These findings are consistent with the EMSA results where IscR only bound to MU2 at 4 μM (Fig 2A). No binding of IscR to MU3 could be detected at any concentration tested. These results suggest that site B is the primary IscR binding site. Binding of IscR to the site B region promotes additional binding of IscR to site A. In the absence of site B binding to site A was detected at a higher concentration (4 μM) of IscR.

In PAO1, the binding characteristic of IscR to its target sites on the *iscR* promoter region reveals another possible mode of IscR regulation of the *isc* operon. We observed protection of the site A at a lower concentration of IscR (1 μM) when site B was also bound, in comparison to when binding at site B was disrupted due to the MU2 mutation of the promoter sequence (4 μM of IscR needed for protection, Fig 2B). These results suggest that IscR binding to site B could facilitate additional IscR binding to the lower-affinity site A, ensuring that IscR occupies both B and A binding sites. However, our current data cannot clearly differentiate between alternative modes of IscR binding, and further studies are needed to refine the mode of PAO1 IscR binding to the *isc* regulatory region. The positions of these Type-1 binding sites in the vicinity of promoter motifs ensure that binding of IscR to these sites would hinder RNA polymerase from binding to the promoter motifs, resulting in repression of *isc* operon expression.

### Two Type-1 sites are required for repressing the *isc* promoter *in vivo*

**Fig 3:**
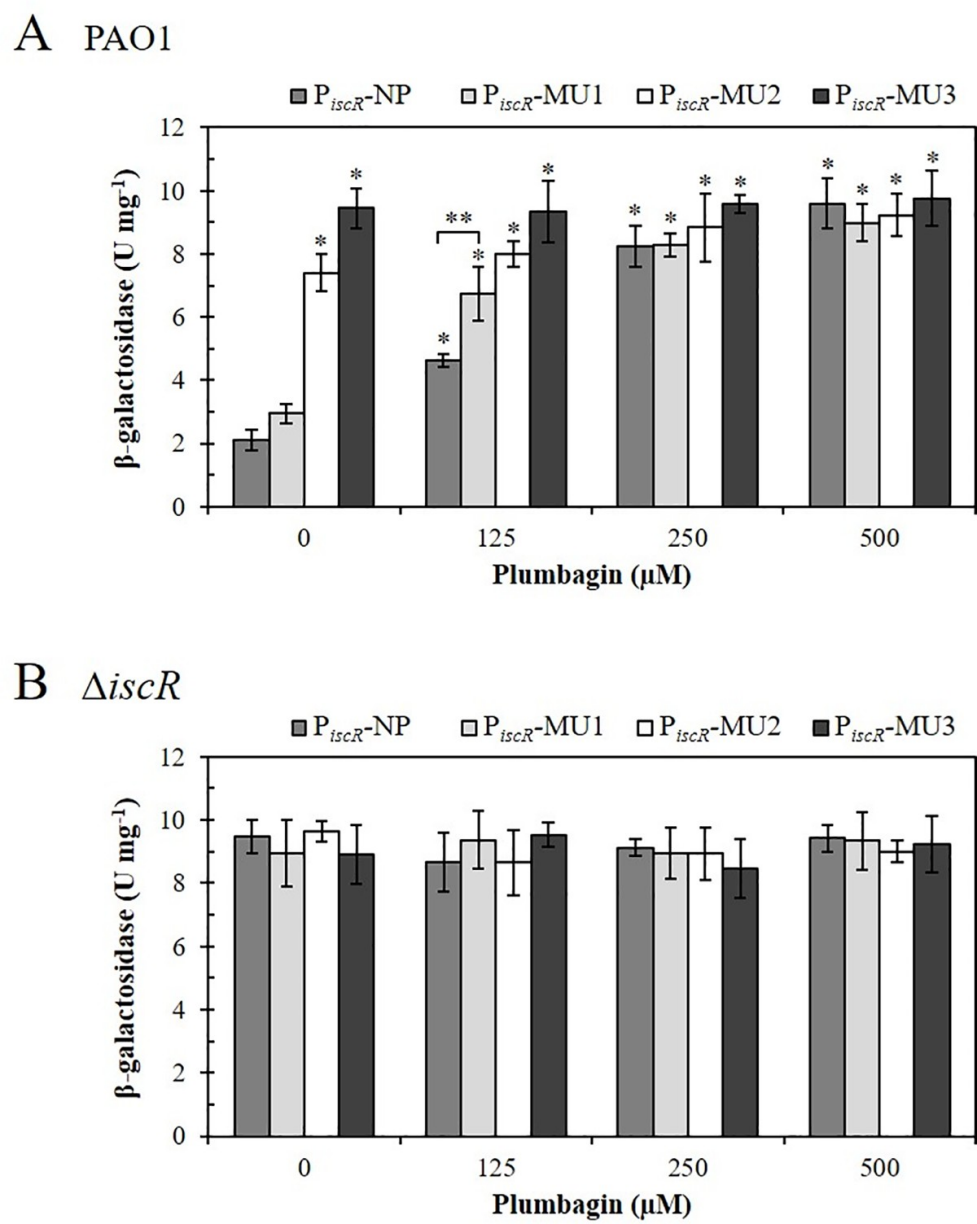
Determination of the mutated *isc* promoter activity *in vivo*. β-galactosidase activity assays were performed to determine the promoter activity of the mutated *isc* promoter in (A) *P*. *aeruginosa* PAO1 and (B) Δ*iscR* mutant strains. The *isc* native (P_*iscR*_-NP) and mutated promoter (P_*iscR*_-MU1, P_*iscR*_-MU2, and P_*iscR*_-MU3) were transcriptionally fused with the *lacZ* reporter gene. Bacterial cultures were treated with 0, 125, 250, and 500 μM Plumbagin (PB). The data shown are the means and standard deviations of three biologically independent experiments. The asterisk indicates a statistically significant difference (*p* < 0.05 by one-way ANOVA with Bonferroni correction) compared to either P_*iscR*_-NP (*) or P_*iscR*_-MU1 (**).

Next, the role of IscR binding to site A and site B of the *iscR* promoter fragment on the repression of the *isc* promoter was investigated using promoter-*lacZ* fusion. The *iscR* native promoter (P_*iscR*_-NP) and MU1, MU2, and MU3 mutagenized promoter fragments (P_*iscR*_-MU1, P_*iscR*_-MU2, and P_*iscR*_-MU3, respectively) were transcriptionally fused with a promoterless β-galactosidase (*lacZ*) gene in pUC18-mini-Tn7T-Gm-*lacZ* [34]. The mini-Tn7T constructs were transposed into PAO1 and Δ*iscR* mutant [13] genomes. Analysis of β-galactosidase activity obtained from cultures of PAO1 and Δ*iscR* containing various P_*iscR*_-*lacZ* treated with 0 to 500 μM Plumbagin (PB, as an inducer of IscR [13]). Plumbagin has been shown to generate reactive oxygen species (ROS) via redox cycling reaction in bacteria. The superoxide anions generated by this reaction can damage [Fe-S]-cluster containing proteins and it has been shown that the expression of *iscR* is highly induced by treatment with Plumbagin (16.8-fold) [13, 39–41]. The results of β-galactosidase activity assays are shown in Fig 3. In PAO1::Tn7T-P_*iscR*_-NP-*lacZ*, where *lacZ* was driven by the *isc* NP promoter, treating the bacteria with PB ranging from 0 to 500 μM resulted in the dose-dependent induction of β-galactosidase activity by 2- to 5-fold (Fig 3A). The PB induction of *lacZ* expression from the promoter was abolished in the Δ*iscR* (*iscR*::Tn7T-P_*iscR*_-NP-*lacZ*) mutant, where the promoter activities specified by the native *iscR* promoter were also constitutively high (Fig 3B). These results confirmed the role of IscR as a repressor of the *isc* promoter during nonstressed conditions and upon exposure to oxidative stress (PB), in which the repressor was inactivated, thereby allowing high levels of gene expression from the promoter. This is consistent with the previously reported RT-PCR gene expression study on the role of IscR as a repressor [13].

The results of the EMSA and DNaseI footprint experiments indicated that IscR specifically bound to site B with a lower concentration of IscR than it bound to site A. In the presence of native site B and mutagenized site A, there was little effect on the overall IscR binding patterns to the *isc* promoter. This raises the question of whether binding site A of the promoter fragment had any *in vivo* roles in IscR regulation of the promoter. The *lacZ* expression driven by the mutated MU1, MU2, and MU3 *isc* promoters (P_*iscR*_-MU1-*lacZ*, P_*iscR*_-MU2-*lacZ*, and P_*iscR*_-MU3-*lacZ*) in PAO1 and the Δ*iscR* mutant were also examined under uninduced and PB-induced conditions. The promoter activity of the mutated site A *isc* promoter (MU1) in PAO1 under uninduced conditions showed slightly higher β-galactosidase activity (2.9 U mg^-1^) than the native promoter (2.1 U mg^-1^). Treatment of the cultures with a low concentration (125 μM) of PB led to a marginally greater magnitude of induction of β-galactosidase activity, specifically, 6.8 U mg^-1^ for the MU1 promoter compared with 4.6 U mg^-1^ for the NP *isc* promoter. However, when cultures were treated with higher concentrations (250 to 500 μM) of PB, both MU1 and NP promoters displayed similar induction patterns and magnitudes of β-galactosidase activity (Fig 3A). In contrast, mutations at the 5’ end (MU2) or the 3’ end (MU3) of site B severely affected the regulation of the *isc* promoter by IscR. In uninduced PAO1, MU2 and MU3 *isc* promoter fragments showed high levels of β-galactosidase activity (7.4 U mg^-1^ and 9.4 U mg^-1^, respectively) compared to the native promoter (2.1 U mg^-1^) (Fig 3A). The levels of β-galactosidase activity specified by MU2 and MU3 *isc* promoters in PAO1 cultures treated with 125 to 500 μM PB were similar to the high level observed in uninduced cultures (Fig 3A). Hence, these site-B-mutated *isc* promoters were responsible for constitutively high levels of *lacZ* expression. The experiments were repeated in the Δ*iscR* mutant containing NP, MU1, MU2, and MU3 *isc* promoters grown under induction with 0 to 500 μM PB. These *isc* promoter-*lacZ* fusion constructs showed constitutively high levels of β-galactosidase activity in the range of 8.5 to 9.5 U mg^-1^.

The PAO1 strain with mutated site A (MU1) promoter showed a small increase in basal promoter activity compared to native promoter (NP), suggesting that IscR binding to mutated site A had a minor effect on the overall IscR repression of the *isc* promoter (Fig 3A). PB treatment of MU1 further enhanced β-galactosidase activities (Fig 3A). This probably due to oxidation of holo-IscR, which reduced its ability to bind to IscR Type-1 binding sites. This reduced binding to the mutated site A led to the increased levels of β-galactosidase activities observed in the MU1 promoter treated with low concentrations of PB. The *in vivo* results showed that the binding of IscR to site A has a minor but significant contribution to overall IscR promoter repression. The *in vivo* effects of mutations at the major IscR binding site B (MU2 and MU3) were constitutively high levels of β-galactosidase activity. It is likely that MU2 that MU3 mutations eliminated IscR binding to the primary binding site B. This notion is supported by DNaseI footprinting results showing that IscR could not bind to MU2 and MU3 promoter fragments (Fig 2B). In the Δ*iscR* mutant, NP, and mutated *isc* promoters showed constitutively high levels of β-galactosidase activity, confirming that the observed repression and PB induction of β-galactosidase activities were mediated by IscR (Fig 3B). Furthermore, the observed constitutively high promoter activity provides evidence that MU1, MU2, and MU3 mutations did not affect promoter activity but affected IscR binding efficiency to its binding sites.

Unlike IscR-regulated genes in other bacteria, the *isc* operon has two IscR binding sites present in the vicinity of the conserved promoter motifs. Two tandem IscR binding sites are often associated with the regulation of genes for [Fe-S] cluster biosynthesis [22, 26, 42], while other IscR target genes have a single IscR binding site.

### [Fe-S] cluster ligated IscR is required for binding to IscR Type-1 binding sites on the *isc* promoter

**Fig 4:**
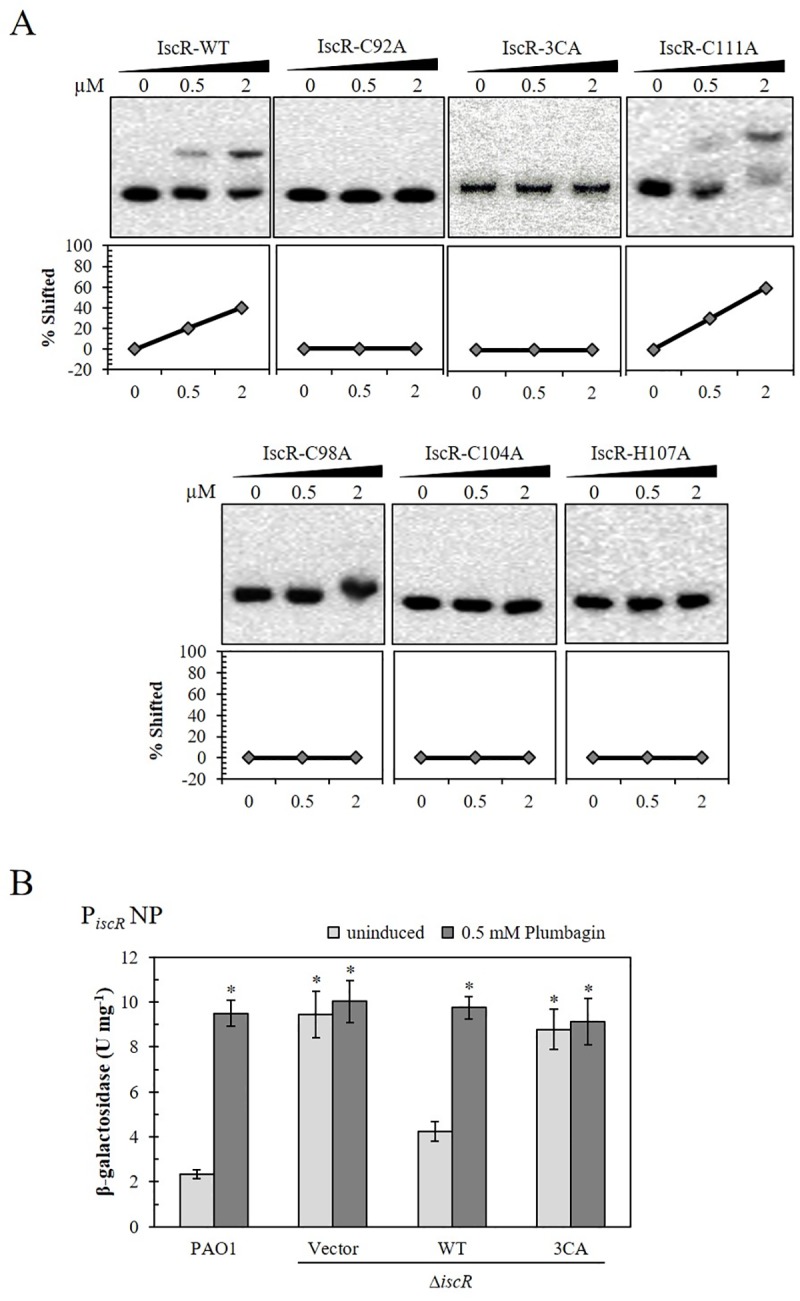
The effect of mutations in [Fe-S] cluster ligation residues of IscR on *in vitro* binding affinity to *isc* promoter and *in vivo isc* promoter activity. (A) An EMSA was performed using purified wild-type IscR (IscR-WT) and mutant IscR proteins (IscR-C92A, IscR-C98A, IscR-C104A, IscR-C111A, IscR-H107A, and IscR-3CA) in which C92, C98, and C104 were changed to A. The percentage of bound probe was calculated from the band intensity and is shown below each EMSA panel. (B) Promoter activity of the *isc* native promoter (P_*iscR*_-NP) was determined in *P*. *aeruginosa* PAO1::Tn7T- P_*iscR*_-WT-*lacZ* harboring empty pBBR1MCS-4 vector (PAO1) and Δ*iscR*::Tn7T- P_*iscR*_-WT-*lacZ* mutant strains harboring plasmids expressing *iscR* (WT), *iscR*-3CA (3CA), and empty pBBR1MCS-4 vector (Vector). The cultures were grown with or without 0.5 mM Plumbagin induction. The data shown are the means and the standard deviations of three biologically independent experiments. The asterisk indicates a statistically significant difference (*p* < 0.05 by one-way ANOVA with Bonferroni correction).

The [Fe-S] cluster of IscR has been shown to be important in sensing stresses and in the control of genes in the IscR regulon [13, 19]. In PAO1, [Fe-S] clusters are important for IscR function as a transcriptional repressor. Mutations that inhibit the cluster ligation of IscRs result in a defect in the repression of target gene expression [25]. Next, purified mutated IscR proteins (C92A, C98A, C104A, H107A, C111A, and 3CA) with native *isc* promoter fragment (NP) were used in the EMSA assay. The purified IscR with a single mutation in [2Fe-2S] cluster-ligating amino acids (C92A, C98A, C104A, and H107A) at concentrations up to 2 μM did not bind to the promoter fragment. Similarly, IscR-3CA also did not bind the promoter fragments (Fig 4A). Both wild-type IscR and IscR C111A (a mutated C111 residue not involved in the cluster ligation [13]) showed significant binding to the promoter fragments even at a concentration of 0.5 μM (Fig 4A). Both IscR proteins had similar binding affinity to the promoter fragment. As expected, IscR-3CA, possibly corresponding to apo-IscR ([21] and in this study), was unable to bind both binding sites on the *isc* promoter region, suggesting that the apo-IscR form could not repress *isc* operon expression and that only [2Fe-2S]-IscR could bind to both binding sites.

The ability of IscR-3CA to repress gene expression *in vivo* was examined in Δ*iscR*::Tn7T-P_*iscR*_-WT-*lacZ*/pBBR-IscR. The expression of *iscR*-WT in Δ*iscR* was able to repress *lacZ* transcription from the *isc* promoter, as shown by decreased β-galactosidase activity (4.3 U mg^-1^) compared with the level attained by Δ*iscR*::Tn7T-P_*iscR*_-WT-*lacZ*/pBBR control (9.5 U mg^-1^). Furthermore, promoter activity could be induced by PB treatment when IscR was expressed compared to the vector control (Fig 4B). The expression of *iscR-*3CA (Δ*iscR*::Tn7T-P_*iscR*_P-WT-*lacZ*/pBBR-IscR-3CA) could not repress promoter activity and showed constitutively high levels of β-galactosidase activity (8.8 U mg^-1^ for uninduced and 9.1 U mg^-1^ for PB-induced) (Fig 4B). This indicates that the [2Fe-2S] cluster ligation is essential for IscR binding on the *isc* promoter *in vivo*. Here, we showed that the defect in repression of gene expression observed for the IscR mutant protein was due to its inability to bind to Type-1 binding sites on the *isc* promoter.

The results support the proposed hypothesis that under nonstressed conditions, [2Fe-2S]-IscR represses *isc* operon expression, while upon exposure to oxidative stress conditions, [2Fe-2S] clusters could be oxidized by ROS, leading to the loss of clusters and formation of apo-IscR, which could not bind the promoter, and resulting in derepression of the *isc* operon.

### Analysis of IscR Type-1 binding sites and the role of [Fe-S]-IscR in the repression of IscR-target gene expression

**Fig 5:**
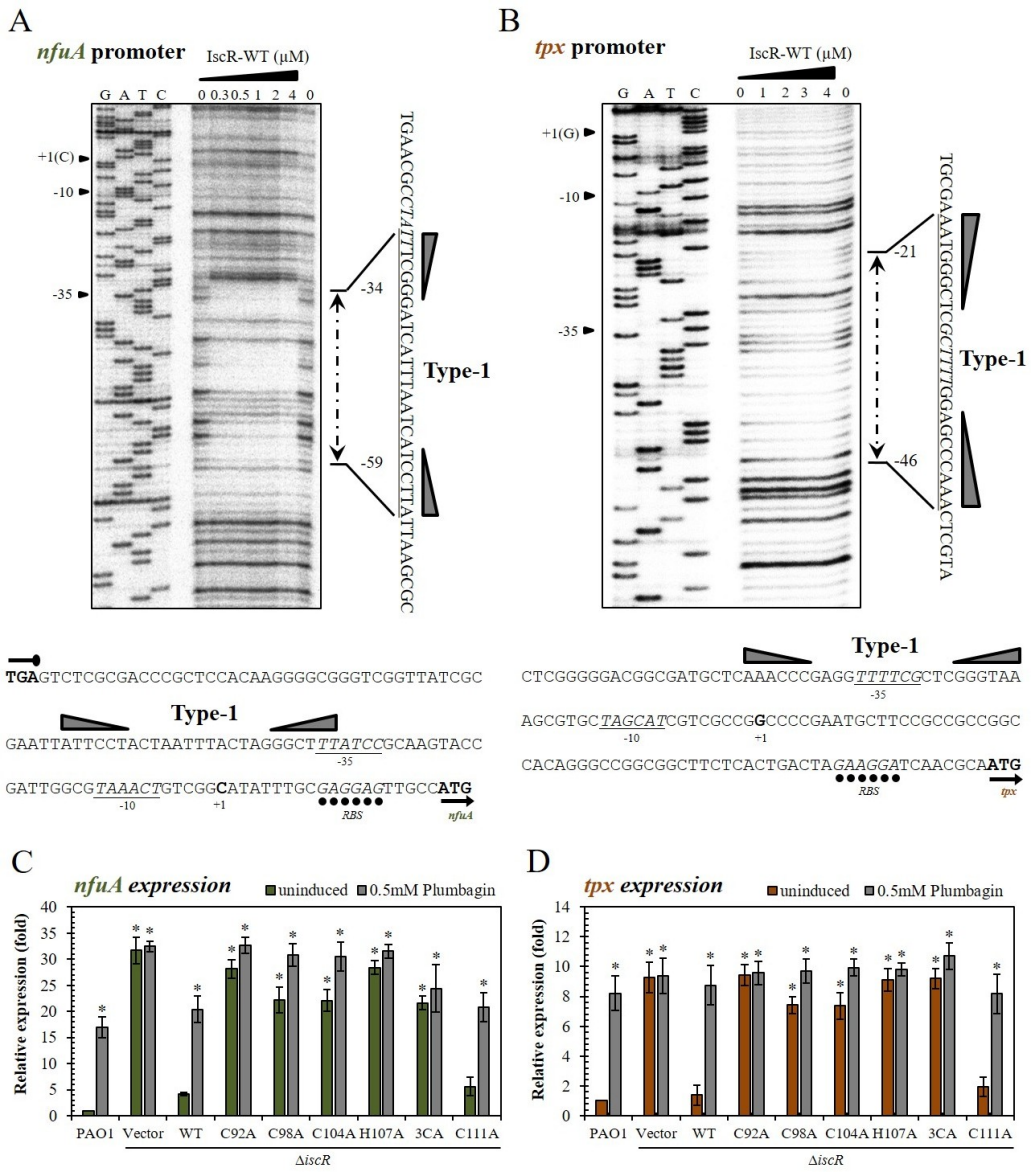
*In vitro* and *in vivo* studies of IscR binding properties and expression on its target gene promoters. DNaseI footprinting experiments were performed using purified wild-type IscR (IscR-WT) and the promoter fragments of *nfuA* (A) or *tpx* (B). Underlined and italic fonts represent the -35 and -10 promoter motifs and ribosome binding site (RBS), respectively. G, A, T, and C represent DNA sequence ladders. The IscR Type-1 binding site is indicated by right and left triangles. The expression levels of *nfuA* (C) and *tpx* (D) were monitored using qRT-PCR. RNA samples were prepared from cultures of *P*. *aeruginosa* PAO1 and the Δ*iscR* mutant harboring plasmid expressing *iscR* (WT), *iscR-*C92A, *iscR-*C98A, *iscR-*C104A, *iscR-*C111A, *iscR-*H107A, *iscR*-3CA, and empty vector control (Vector). The cultures were grown with or without 0.5 mM Plumbagin induction. The data shown are the means and the standard deviations of three biologically independent experiments. The asterisk indicates a statistically significant difference (*p* < 0.05 by one-way ANOVA with Bonferroni correction).

IscR is a global transcription regulator [13, 19–21]. Genes in the IscR regulon play important roles in bacterial cells under both physiological and stressful environments. In PAO1, IscR was previously shown by EMSAs to directly regulated *nfuA* and *tpx* through direct binding to the promoters [5, 25]. However, the IscR binding DNA sequences and the nature of IscR involved in binding to the promoter regions of these genes have not been investigated. The DNaseI footprinting assay was performed using IscR and promoter fragments from the *nfuA* and *tpx* genes. The binding of IscR to putative IscR Type-1 binding sites in the vicinity of *nfuA* and *tpx* promoters that are responsible for repression of gene expression was examined. The DNaseI footprinting results in Fig 5A and 5B show the protected regions at positions −59 to −34 (5’ATTCCTACTAATTTACTAGGGCTTT3’) and −46 to −21 (5’AAACCCGAGGTTTTCGCTCGGGTAA3’) for the *nfuA* and *tpx* promoters, respectively. The patterns of IscR binding to *nfuA* and *tpx* promoter fragments showed only a single IscR binding site on each promoter fragment. The sequences of the protected regions shared a high degree of identity (64% for *nfuA* and 72% for *tpx*) with the conserved *E*. *coli* IscR Type-1 binding site. These IscR Type-1 binding sites overlapped the -35 motif of the *nfuA* promoter and covered the -35 motif of the *tpx* promoter (Fig 5A and 5B). Binding of IscR to Type-1 sites of these genes would hinder RNA polymerase from binding to the promoters, leading to repression of gene expression. The results are in agreement with the *in vivo nfuA* and *tpx* expression analysis, where IscR functions as a repressor of these genes [5, 25], and supports the proposed role of IscR as a repressor once it binds to Type-1 binding sites.

We have shown that [2Fe-2S]-IscR is an active form of the regulator that binds to the Type-1 binding site. Hence, the role of [Fe-S] cluster ligation of IscR on *nfuA* and *tpx* expression was evaluated using the mutated IscR-3CA, in which 3 cysteine residues involved in [2Fe-2S] cluster ligation were mutated. The Δ*iscR* mutant harboring *iscR*-WT or *iscR-*3CA expression plasmids and PAO1 control were grown either uninduced or induced with 0.5 mM PB, and the *nfuA* and *tpx* expression levels were monitored. As expected, in PAO1, the expression of both *nfuA* and *tpx* was highly induced by PB treatment (Fig 5C and 5D). In the Δ*iscR* mutant, the expression of both genes was constitutively high and PB-induced gene expression was abolished (Fig 5C and 5D), and the altered phenotypes could be complemented in the Δ*iscR* mutant harboring *iscR-*WT (Fig 5C and 5D). In contrast, the Δ*iscR* mutant harboring *iscR-*3CA showed constitutively high levels of expression in both genes under uninduced and PB-induced conditions (Fig 5C and 5D). The results indicated that the [2Fe-2S] cluster ligated IscR was required for the IscR repression of target gene expression. These findings are consistent with *in vitro* observations that indicate cluster-ligated IscR is essential for IscR binding to the Type-1 binding site, resulting in repression of gene expression (Fig 5C and 5D, [21]).

### Identification of IscR Type-1 binding sites

**Fig 6:**
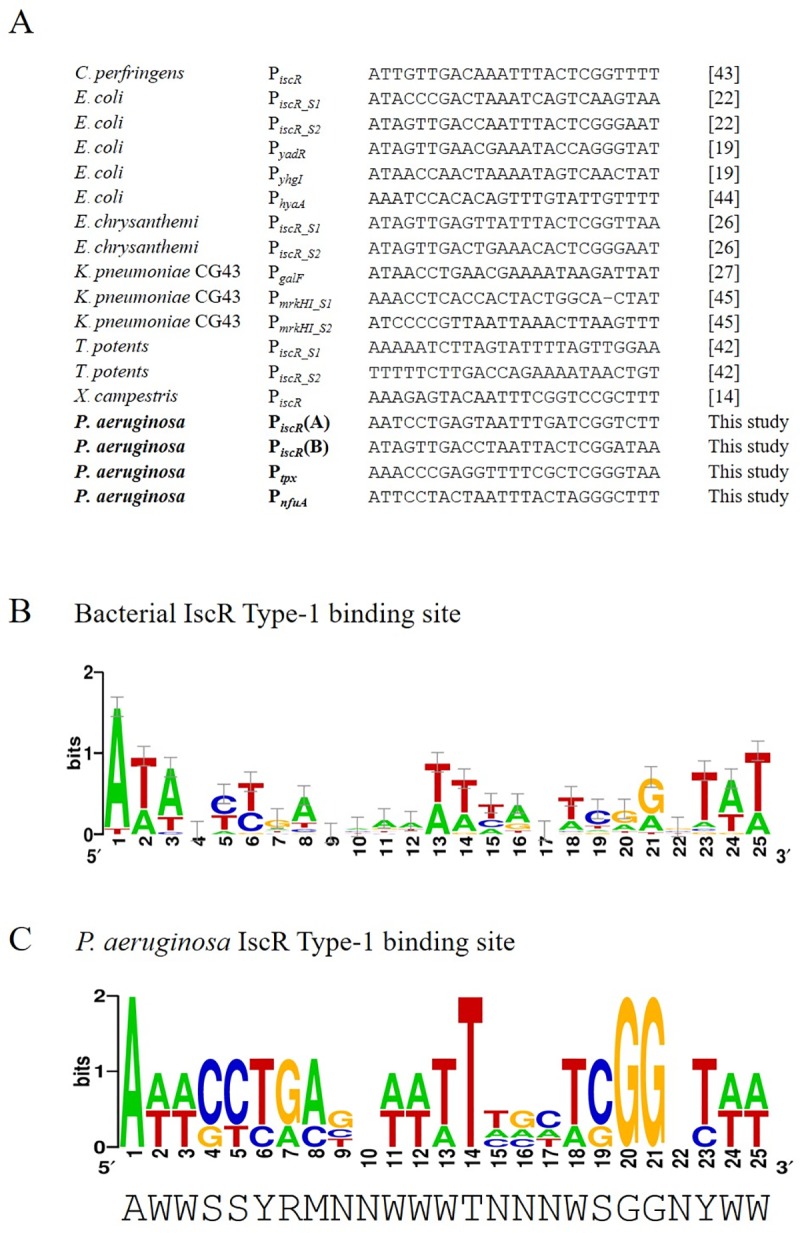
Comparison of IscR binding motifs identified within the *P*. *aeruginosa* PAO1 genome and among various bacterial species. (A) Multiple alignment of the known IscR Type-1 binding sequences among various bacteria, including *C*. *perfringens*, *E*. *coli*, *E*. *chrysanthemi*, *K*. *pneumoniae*, *T*. *potens*, and *X*. *campestris*, and within the *P*. *aeruginosa* PAO1 genome, including *iscR* (sites A and B), *nfuA*, and *tpx* promoters were computationally compared with the bold text. Square brackets indicate the reference. The proposed logo sequences of the bacterial (B) and *P*. *aeruginosa* PAO1 (C) IscR Type-1 binding sites were constructed using WebLogo [46].

The ability to identify IscR Type-1 binding sites in bacterial genomes is an important tool for the analysis of IscR global regulation of gene expression. The availability of characterized IscR Type-1 binding sites from different bacteria, including *Clostridium perfringens* [43], *E*. *coli* [19, 20, 22, 44], *Erwinia chrysanthemi* [26], *Klebsiella pneumoniae* [27, 45], *Thylacinus potens* [42], *Xanthomonas campestris* [14], and *P*. *aeruginosa* (this study), have allowed the generation of an IscR Type-1 binding site sequence logo (Fig 6A and 6B). This sequence logo could be used for the identification and characterization of new IscR Type-1 binding site-containing genes.

The availability of characterized IscR Type-1 binding sites of genes (Site A and Site B *iscR*, *nfuA*, and *tpx*) in *P*. *aeruginosa* has allowed us to perform a more refined determination of IscR Type-1 binding motif sequence logo using WebLogo online bioinformatics tools [46]. The proposed *P*. *aeruginosa* IscR Type-1 binding site sequence logo is 5’AWWSSYRMNNWWWTNNNWSGGNYWW3’, which is expressed in the format according to the IUPAC nucleotide code (Fig 6C). There are some sequence differences in Type-1 sequence logos; CCTGA at positions 4–8, T at position 14 and GG at positions 20 and 21 have high prevalence in *P*. *aeruginosa* but not in other bacteria.

## Conclusion

**Fig 7:**
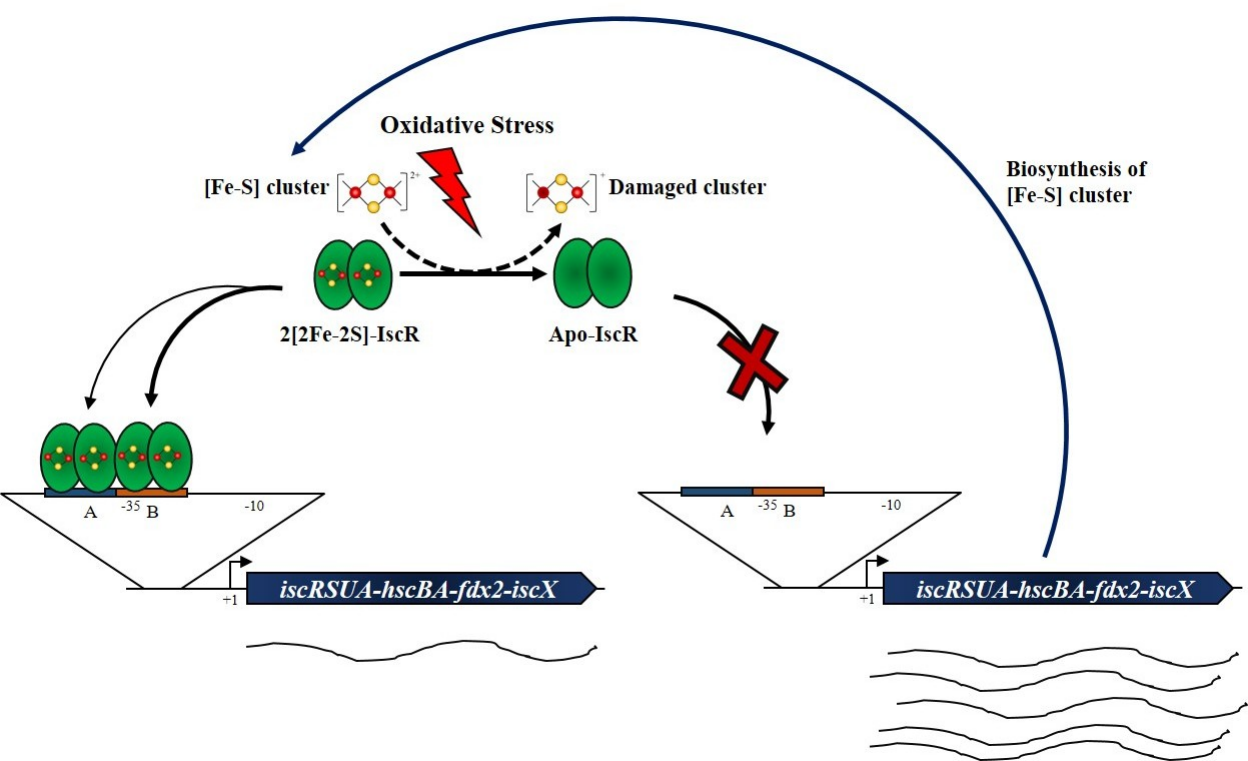
Proposed model for IscR-regulated *isc* expression under oxidative stress conditions in *P*. *aeruginosa*. Under normal physiological conditions, [2Fe-2S] cluster-containing IscR binds two Type-1 binding sites in a stepwise manner. Upon exposure to oxidative stress conditions, ROS react and damage cellular [Fe-S] clusters, resulting in the loss of clusters from [Fe-S] cluster-containing proteins, including IscR, leading to derepression of *isc* operon expression to rebuild the active [Fe-S] clusters.

In *P*. *aeruginosa*, [2Fe-2S] cluster-ligated IscR is the active form of the regulator required for binding the Type-1 binding sites, resulting in repression of gene expression. We proposed that *P*. *aeruginosa* IscR could have different modes of binding to target sites. IscR binds to the Type-1 binding sites on the *isc* promoter region primarily at lower concentrations to site B and subsequently at higher concentrations to site A by either a stepwise or a cooperative mode of binding. Although, our data could not differentiate other alternative IscR binding modes, the mode of IscR binding to Type-1 sites and the number of binding sites involved in the regulation of the *isc* operon in *P*. *aeruginosa* are unlike the previously characterized *E*. *coli* model of IscR regulation of the operon, where IscR independently bound to either site [21]. The distinction in mode of IscR binding between PAO1 and *E*. *coli* could arise from their physiological differences, where the PAO1 genome only contains the *isc* operon and lacks the redundant *suf* and *nif* gene clusters [18]. The location of these Type-1 binding sites in the vicinity of the promoter provides the link between binding of the transcription regulator and gene expression. Under normal growth, IscR occupies both site B and A binding sites and hinders RNA polymerase binding to the promoter, resulting in repression of *isc* operon expression. This reduces the levels of enzymes available for the biosynthesis of [Fe-S] clusters and decreases new cluster synthesis and assembly. Upon exposure to inducing conditions, such as oxidative stress, higher levels of ROS react with and subsequently damage [Fe-S] clusters (Fig 7). This could result in the loss of clusters in the [Fe-S] cluster-containing proteins and/or prevent damaged clusters from being incorporated into the proteins. The presence of dual IscR binding sites with different binding affinities in the vicinity of the promoter probably affords more refined control of gene expression and rapid response under changing [Fe-S] cluster status of the bacteria. This could contribute to the dynamics in the transcriptional control of IscR under different levels of stress exposure.

Effectively, this would lower the levels of [Fe-S] cluster-ligated IscR, resulting in loss of the repressor binding to the Type-1 binding sites. This allows RNA polymerase to bind to promoter motifs and derepresses the *isc* operon expression. Analysis of additional IscR Type-1 binding sites (*nfuA* and *tpx*) show that the location of the binding sites is in close proximity to the promoter motifs where [Fe-S] cluster-ligated IscR binding would prevent binding of RNA polymerase to promoters, resulting in repression of gene expression. Hence, IscR most likely indirectly senses the levels of [Fe-S] clusters where changes in cluster levels would reflect in the level of [Fe-S] cluster-ligated IscR, which globally determines the degree of binding to Type-1 sites and repression of target genes. Ultimately, we proposed *P*. *aeruginosa* IscR Type-1 binding site sequence logo as 5’AWWSSYRMNNWWWTNNNWSGGNYWW3’, which could be useful for the identification of novel IscR Type-1 regulated genes, contributing to the understanding of the roles of an [Fe-S] cluster in diverse cellular activities.

## Supporting information

S1 File UV-visible absorption spectra of IscR-3CA comparing to IscR-WT. UV-visible absorption spectra of IscR-WT and IscR-3CA with or without 0.05 M H_2_O_2_ treatment was determined. Purified IscR proteins (10 μM) in 50 mM phosphate buffer (PB) were used in the experiments. PB was used as a control.
(DOCX)

S1 Table Raw data behind the *in vitro* characterization of the mutated *isc* promoter (Fig 3). (XLSX)

S2 Table Raw data behind the effect of mutations in [Fe-S] cluster ligation residues of IscR on *in vitro* binding affinity to *isc* promoter and *in vivo isc* promoter activity (Fig 4B). (XLSX)

S3 Table Raw data behind the *in vitro* and *in vivo* studies of IscR binding properties and expression on its target gene promoters (Fig 5C and 5D). (XLSX)

## References

[1] Vasil ML, Ochsner UA. The response of *Pseudomonas aeruginosa* to iron: genetics, biochemistry and virulence. Mol Microbiol. 1999; 34(3):399–413. . 10564483 10.1046/j.1365-2958.1999.01586.x

[2] Romsang A, Atichartpongkul S, Trinachartvanit W, Vattanaviboon P, Mongkolsuk S. Gene expression and physiological role of *Pseudomonas aeruginosa* methionine sulfoxide reductases during oxidative stress. J Bacteriol. 2013; 195(15):3299–308. 10.1128/JB.00167-13 . 23687271 PMC3719549

[3] Romsang A, Duang-Nkern J, Khemsom K, Wongsaroj L, Saninjuk K, Fuangthong M, et al Pseudomonas aeruginosa ttcA encoding tRNA-thiolating protein requires an iron-sulfur cluster to participate in hydrogen peroxide-mediated stress protection and pathogenicity. Sci Rep. 2018; 8(1):11882 10.1038/s41598-018-30368-y . 30089777 PMC6082896

[4] Romsang A, Duang-Nkern J, Wirathorn W, Vattanaviboon P, Mongkolsuk S. Pseudomonas aeruginosa IscR-regulated ferredoxin NADP(+) reductase gene (fprB) functions in iron-sulfur cluster biogenesis and multiple stress response. PLoS One. 2015; 10(7):e0134374 10.1371/journal.pone.0134374 . 26230408 PMC4521836

[5] Somprasong N, Jittawuttipoka T, Duang-Nkern J, Romsang A, Chaiyen P, Schweizer HP, et al *Pseudomonas aeruginosa* thiol peroxidase protects against hydrogen peroxide toxicity and displays atypical patterns of gene regulation. J Bacteriol. 2012; 194(15):3904–12. 10.1128/JB.00347-12 . 22609922 PMC3416540

[6] Wongsaroj L, Saninjuk K, Romsang A, Duang-Nkern J, Trinachartvanit W, Vattanaviboon P, et al Pseudomonas aeruginosa glutathione biosynthesis genes play multiple roles in stress protection, bacterial virulence and biofilm formation. PLoS One. 2018; 13(10):e0205815 10.1371/journal.pone.0205815 . 30325949 PMC6191110

[7] Py B, Barras F. Building Fe-S proteins: bacterial strategies. Nat Rev Microbiol. 2010; 8(6):436–46. 10.1038/nrmicro2356 . 20467446

[8] Santos JA, Pereira PJ, Macedo-Ribeiro S. What a difference a cluster makes: The multifaceted roles of IscR in gene regulation and DNA recognition. Biochim Biophys Acta. 2015; 1854(9):1101–12. 10.1016/j.bbapap.2015.01.010 . 25641558

[9] Kimura S, Suzuki T. Iron-sulfur proteins responsible for RNA modifications. Biochim Biophys Acta. 2015; 1853(6):1272–83. 10.1016/j.bbamcr.2014.12.010 . 25533083

[10] Roche B, Aussel L, Ezraty B, Mandin P, Py B, Barras F. Reprint of: Iron/sulfur proteins biogenesis in prokaryotes: formation, regulation and diversity. Biochim Biophys Acta. 2013; 1827(8–9):923–37. 10.1016/j.bbabio.2013.05.001 . 23660107

[11] Crack JC, Stapleton MR, Green J, Thomson AJ, Le Brun NE. Influence of association state and DNA binding on the O_2_-reactivity of [4Fe-4S] fumarate and nitrate reduction (FNR) regulator. Biochem J. 2014; 463(1):83–92. 10.1042/BJ20140169 . 25019503 PMC4214427

[12] Lushchak OV, Piroddi M, Galli F, Lushchak VI. Aconitase post-translational modification as a key in linkage between Krebs cycle, iron homeostasis, redox signaling, and metabolism of reactive oxygen species. Redox Rep. 2014; 19(1):8–15. 10.1179/1351000213Y.0000000073 . 24266943 PMC6837700

[13] Romsang A, Duang-Nkern J, Leesukon P, Saninjuk K, Vattanaviboon P, Mongkolsuk S. The iron-sulphur cluster biosynthesis regulator IscR contributes to iron homeostasis and resistance to oxidants in Pseudomonas aeruginosa. PLoS One. 2014; 9(1):e86763 10.1371/journal.pone.0086763 . 24466226 PMC3899308

[14] Fuangthong M, Jittawuttipoka T, Wisitkamol R, Romsang A, Duang-nkern J, Vattanaviboon P, et al IscR plays a role in oxidative stress resistance and pathogenicity of a plant pathogen, *Xanthomonas campestris*. Microbiol Res. 2015; 170:139–46. 10.1016/j.micres.2014.08.004 . 25200360

[15] Outten FW. Recent advances in the Suf Fe-S cluster biogenesis pathway: Beyond the Proteobacteria. Biochim Biophys Acta. 2015; 1853(6):1464–9. 10.1016/j.bbamcr.2014.11.001 . 25447545 PMC4390423

[16] Tanaka N, Kanazawa M, Tonosaki K, Yokoyama N, Kuzuyama T, Takahashi Y. Novel features of the ISC machinery revealed by characterization of *Escherichia coli* mutants that survive without iron-sulfur clusters. Mol Microbiol. 2016; 99(5):835–48. 10.1111/mmi.13271 . 26560204

[17] Lee KC, Yeo WS, Roe JH. Oxidant-responsive induction of the suf operon, encoding a Fe-S assembly system, through Fur and IscR in *Escherichia coli*. J Bacteriol. 2008; 190(24):8244–7. 10.1128/JB.01161-08 . 18849427 PMC2593202

[18] Romsang A, Dubbs J, Mongkolsuk S. The iron-sulfur cluster biosynthesis regulator IscR contributes to iron homeostasis and resistance to oxidants in Pseudomonas aeruginosa In: IdB FJ, editor. Stress and Environmental Regulation of Gene Expression and Adaptation in Bacteria. 2 John Wiley & Sons, USA: John Wiley & Sons; 2016 p. 1090–102.

[19] Giel JL, Rodionov D, Liu M, Blattner FR, Kiley PJ. IscR-dependent gene expression links iron-sulphur cluster assembly to the control of O_2_-regulated genes in *Escherichia coli*. Mol Microbiol. 2006; 60(4):1058–75. 10.1111/j.1365-2958.2006.05160.x . 16677314

[20] Nesbit AD, Giel JL, Rose JC, Kiley PJ. Sequence-specific binding to a subset of IscR-regulated promoters does not require IscR Fe-S cluster ligation. J Mol Biol. 2009; 387(1):28–41. 10.1016/j.jmb.2009.01.055 . 19361432 PMC2709974

[21] Rajagopalan S, Teter SJ, Zwart PH, Brennan RG, Phillips KJ, Kiley PJ. Studies of IscR reveal a unique mechanism for metal-dependent regulation of DNA binding specificity. Nat Struct Mol Biol. 2013; 20(6):740–7. 10.1038/nsmb.2568 . 23644595 PMC3676455

[22] Giel JL, Nesbit AD, Mettert EL, Fleischhacker AS, Wanta BT, Kiley PJ. Regulation of iron-sulphur cluster homeostasis through transcriptional control of the Isc pathway by [2Fe-2S]-IscR in *Escherichia coli*. Mol Microbiol. 2013; 87(3):478–92. 10.1111/mmi.12052 . 23075318 PMC4108476

[23] Yeo WS, Lee JH, Lee KC, Roe JH. IscR acts as an activator in response to oxidative stress for the suf operon encoding Fe-S assembly proteins. Mol Microbiol. 2006; 61(1):206–18. 10.1111/j.1365-2958.2006.05220.x . 16824106

[24] Miller HK, Kwuan L, Schwiesow L, Bernick DL, Mettert E, Ramirez HA, et al IscR is essential for yersinia pseudotuberculosis type III secretion and virulence. PLoS Pathog. 2014; 10(6):e1004194 10.1371/journal.ppat.1004194 . 24945271 PMC4055776

[25] Romsang A, Duang-Nkern J, Saninjuk K, Vattanaviboon P, Mongkolsuk S. *Pseudomonas aeruginosa nfuA*: Gene regulation and its physiological roles in sustaining growth under stress and anaerobic conditions and maintaining bacterial virulence. PLoS One. 2018; 13(8):e0202151 10.1371/journal.pone.0202151 . 30092083 PMC6084964

[26] Rincon-Enriquez G, Crete P, Barras F, Py B. Biogenesis of Fe/S proteins and pathogenicity: IscR plays a key role in allowing *Erwinia chrysanthemi* to adapt to hostile conditions. Mol Microbiol. 2008; 67(6):1257–73. 10.1111/j.1365-2958.2008.06118.x . 18284573

[27] Wu CC, Wang CK, Chen YC, Lin TH, Jinn TR, Lin CT. IscR regulation of capsular polysaccharide biosynthesis and iron-acquisition systems in Klebsiella pneumoniae CG43. PLoS One. 2014; 9(9):e107812 10.1371/journal.pone.0107812 . 25237815 PMC4169559

[28] Choi KH, Schweizer HP. mini-Tn7 insertion in bacteria with single attTn7 sites: example Pseudomonas aeruginosa. Nat Protoc. 2006; 1(1):153–61. 10.1038/nprot.2006.24 . 17406227

[29] Kovach ME, Phillips RW, Elzer PH, Roop RM 2nd, Peterson KM. pBBR1MCS: a broad-host-range cloning vector. Biotechniques. 1994; 16(5):800–2. . 8068328

[30] Quenee L, Lamotte D, Polack B. Combined *sacB*-based negative selection and *cre-lox* antibiotic marker recycling for efficient gene deletion in *Pseudomonas aeruginosa*. Biotechniques. 2005; 38(1):63–7. 10.2144/05381ST01 . 15679087

[31] Sambrook J, Russell DW. Molecular cloning: a laboratory manual Cold Spring Harbor, New York: Cold Spring Harbor Laboratory Press; 2001.

[32] Choi KH, Kumar A, Schweizer HP. A 10-min method for preparation of highly electrocompetent *Pseudomonas aeruginosa* cells: application for DNA fragment transfer between chromosomes and plasmid transformation. J Microbiol Methods. 2006; 64(3):391–7. 10.1016/j.mimet.2005.06.001 . 15987659

[33] Romsang A, Leesukon P, Duangnkern J, Vattanaviboon P, Mongkolsuk S. Mutation of the gene encoding monothiol glutaredoxin (GrxD) in Pseudomonas aeruginosa increases its susceptibility to polymyxins. Int J Antimicrob Agents. 2015; 45(3):314–8. 10.1016/j.ijantimicag.2014.10.024 . 25593012

[34] Boonma S, Romsang A, Duang-Nkern J, Atichartpongkul S, Trinachartvanit W, Vattanaviboon P, et al The FinR-regulated essential gene fprA, encoding ferredoxin NADP+ reductase: Roles in superoxide-mediated stress protection and virulence of Pseudomonas aeruginosa. PLoS One. 2017; 12(2):e0172071 10.1371/journal.pone.0172071 . 28187184 PMC5302815

[35] Dokpikul T, Chaoprasid P, Saninjuk K, Sirirakphaisarn S, Johnrod J, Nookabkaew S, et al Regulation of the Cobalt/Nickel Efflux Operon *dmeRF* in *Agrobacterium tumefaciens* and a Link between the Iron-Sensing Regulator RirA and Cobalt/Nickel Resistance. Appl Environ Microbiol. 2016; 82(15):4732–42. 10.1128/AEM.01262-16 . 27235438 PMC4984278

[36] Panmanee W, Charoenlap N, Atichartpongkul S, Mahavihakanont A, Whiteside MD, Winsor G, et al The OxyR-regulated phnW gene encoding 2-aminoethylphosphonate:pyruvate aminotransferase helps protect Pseudomonas aeruginosa from tert-butyl hydroperoxide. PLoS One. 2017; 12(12):e0189066 10.1371/journal.pone.0189066 . 29216242 PMC5720770

[37] Daung-nkern J, Vattanaviboon P, Mongkolsuk S. Inactivation of *nfuA* enhances susceptibility of *Pseudomonas aeruginosa* to fluoroquinolone antibiotics. J Antimicrob Chemother. 2010; 65(8):1831–2. 10.1093/jac/dkq194 . 20525732

[38] Winsor GL, Griffiths EJ, Lo R, Dhillon BK, Shay JA, Brinkman FS. Enhanced annotations and features for comparing thousands of *Pseudomonas* genomes in the Pseudomonas genome database. Nucleic Acids Res. 2016; 44(D1):D646–53. 10.1093/nar/gkv1227 . 26578582 PMC4702867

[39] Gardner PR, Fridovich I. Inactivation-reactivation of aconitase in *Escherichia coli*. A sensitive measure of superoxide radical. J Biol Chem. 1992; 267(13):8757–63. . 1315737

[40] Hassan HM, Fridovich I. Intracellular production of superoxide radical and of hydrogen peroxide by redox active compounds. Arch Biochem Biophys. 1979; 196(2):385–95. . 225995 10.1016/0003-9861(79)90289-3

[41] Lin CN, Syu WJ, Sun WS, Chen JW, Chen TH, Don MJ, et al A role of *ygfZ* in the *Escherichia coli* response to plumbagin challenge. J Biomed Sci. 2010; 17:84 10.1186/1423-0127-17-84 . 21059273 PMC2989944

[42] Santos JA, Alonso-Garcia N, Macedo-Ribeiro S, Pereira PJ. The unique regulation of iron-sulfur cluster biogenesis in a Gram-positive bacterium. Proc Natl Acad Sci U S A. 2014; 111(22):E2251–60. 10.1073/pnas.1322728111 . 24847070 PMC4050560

[43] Andre G, Haudecoeur E, Courtois E, Monot M, Dupuy B, Rodionov DA, et al Cpe1786/IscR of *Clostridium perfringens* represses expression of genes involved in Fe-S cluster biogenesis. Res Microbiol. 2017; 168(4):345–55. 10.1016/j.resmic.2016.03.002 . 27020244

[44] Nesbit AD, Fleischhacker AS, Teter SJ, Kiley PJ. ArcA and AppY antagonize IscR repression of hydrogenase-1 expression under anaerobic conditions, revealing a novel mode of O_2_ regulation of gene expression in *Escherichia coli*. J Bacteriol. 2012; 194(24):6892–9. 10.1128/JB.01757-12 . 23065979 PMC3510566

[45] Lin TH, Tseng CY, Lai YC, Wu CC, Huang CF, Lin CT. IscR Regulation of Type 3 Fimbriae Expression in Klebsiella pneumoniae CG43. Front Microbiol. 2017; 8:1984 10.3389/fmicb.2017.01984 . 29085346 PMC5650617

[46] Crooks GE, Hon G, Chandonia JM, Brenner SE. WebLogo: a sequence logo generator. Genome Res. 2004; 14(6):1188–90. 10.1101/gr.849004 . 15173120 PMC419797

