# Supplementary material for: Transcriptional regulation of the Pseudomonas aeruginosa iron-sulfur cluster assembly pathway by binding of IscR to multiple sites
Source: PLoS One. 2019 Jun 28;14(6):e0218385. doi: 10.1371/journal.pone.0218385 (PMC6599224; doi:10.1371/journal.pone.0218385)
Supplement: S1 File — UV-visible absorption spectra of IscR-WT and IscR-3CA with or without 0.05 M H2O2 treatment was determined. Purified IscR proteins (10 μM) in 50 mM phosphate buffer (PB) were used in the experiments. PB was used as a control. (DOCX) [file pone.0218385.s001.docx]

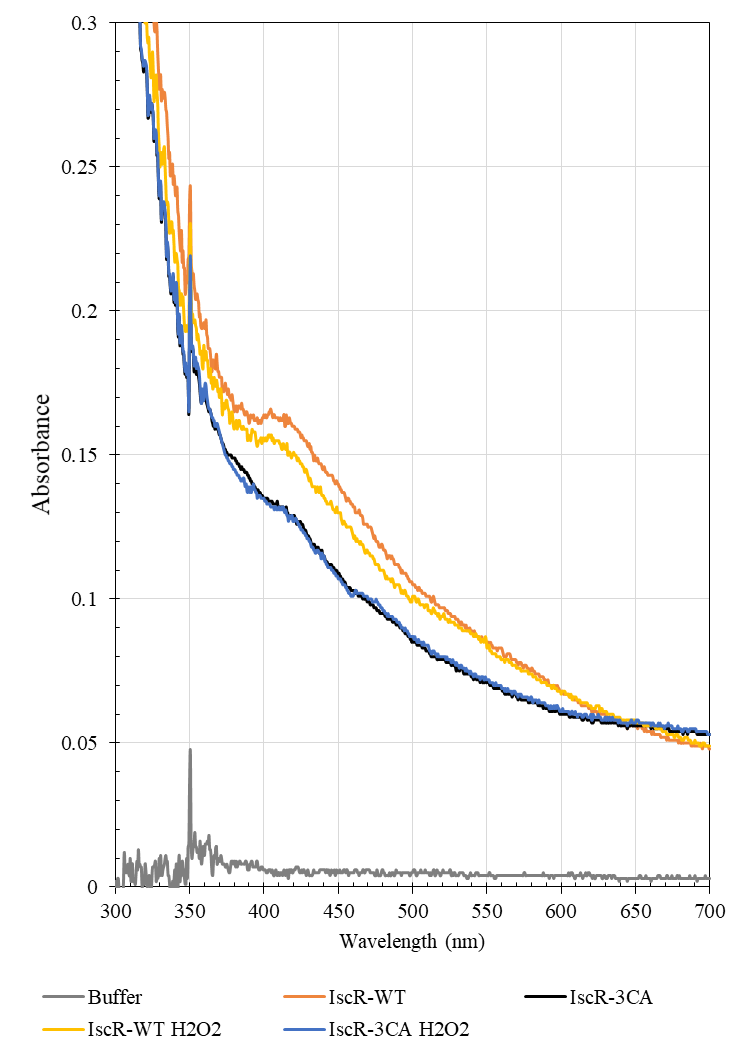


0.05M H_2_O_2_

0.05M H_2_O_2_

**S1 File. UV-visible absorption spectra of IscR-3CA comparing to IscR-WT.** UV-visible absorption spectra of IscR-WT and IscR-3CA treated with or without 0.05 M H_2_O_2_ was determined. Purified IscR proteins in 50 mM phosphate buffer (10 µM) were used in the experiments. 50 mM phosphate buffer was used as control.
